# Multifunctional metaoptics based on bilayer metasurfaces

**DOI:** 10.1038/s41377-019-0193-3

**Published:** 2019-09-04

**Authors:** You Zhou, Ivan I. Kravchenko, Hao Wang, Hanyu Zheng, Gong Gu, Jason Valentine

**Affiliations:** 1 Interdisciplinary Materials Science Program, Vanderbilt University, Nashville, TN 37212 USA; 2 Center for Nanophase Materials Sciences, Oak Ridge National Laboratory, Oak Ridge, TN 37831 USA; 3 Min H. Kao Department of Electrical Engineering and Computer Science, University of Tennessee, Knoxville, TN 37996 USA; 4 Department of Electric Engineering and Computer Science, Vanderbilt University, Nashville, TN 37212 USA; 5 Department of Mechanical Engineering, Vanderbilt University, Nashville, TN 37212 USA

**Keywords:** Metamaterials, Nanophotonics and plasmonics

## Abstract

Optical metasurfaces have become versatile platforms for manipulating the phase, amplitude, and polarization of light. A platform for achieving independent control over each of these properties, however, remains elusive due to the limited engineering space available when using a single-layer metasurface. For instance, multiwavelength metasurfaces suffer from performance limitations due to space filling constraints, while control over phase and amplitude can be achieved, but only for a single polarization. Here, we explore bilayer dielectric metasurfaces to expand the design space for metaoptics. The ability to independently control the geometry and function of each layer enables the development of multifunctional metaoptics in which two or more optical properties are independently designed. As a proof of concept, we demonstrate multiwavelength holograms, multiwavelength waveplates, and polarization-insensitive 3D holograms based on phase and amplitude masks. The proposed architecture opens a new avenue for designing complex flat optics with a wide variety of functionalities.

## Metasurfaces: Bilayers allow more complex control over light

The ability to manipulate light using metasurfaces - surfaces patterned at scales shorter than the wavelengths of the interacting light – can be significantly enhanced using bilayer metasurfaces rather than single layers. Metasurfaces are one of the most active areas of current optical research. Exploring their potential, however, has been restricted by the limitations of single-surface materials. Researchers in the USA, led by Jason Valentine at Vanderbilt University in Tennessee, demonstrate the potential of silicon-based metasurface bilayers for independently controlling the phase, amplitude and polarization of light. Each layer is designed and engineered to manipulate transmitted light differently. This proof of concept work opens a new avenue for creating layered flat and thin materials with complex optical properties. Potential applications should include sophisticated holographic systems and improved methods for optical sensing and optical computing.

## Introduction

Optical metasurfaces are planar nanostructured devices that provide versatile platforms for manipulating the wavefront of light with subwavelength resolution^1,2^. Several configurations have been demonstrated for controlling the amplitude^3–5^, phase^1,6,7^, and polarization^8,9^ of light. Due to their thin form factor and compatibility with integrated circuit fabrication, metasurface lenses^10,11^, beam splitters^12,13^, and waveplates^8,9^ could be attractive replacements for conventional optical elements in certain applications. While success has been achieved in thickness reduction relative to traditional refractive elements, designing metasurfaces with independent control over phase, amplitude, and polarization remains a challenge. It is also desirable to control these properties over broad bandwidths or at multiple wavelengths. While complete control over phase and polarization at a particular wavelength has been demonstrated^14^, independent control of phase and amplitude^15–18^ has been achieved only at the expense of a polarization-dependent response. Several approaches have been investigated to achieve multiwavelength phase control using spatially multiplexed resonators^19–24^, but this generally leads to efficiency degradation due to space filling limitations and resonator cross-talk. Ultimately, the limited design freedom associated with a single resonator layer imposes limits on device functionality.

In this work, we explore a bilayer metasurface architecture with the goal of increasing the design space for metaoptics. We have previously used this architecture to realize multiwavelength lenses using layers composed solely of nanopost resonators^25^. Here, we use bilayers that comprise various combinations of nanoposts, nanodisks, and rectangular nanopillars to increase the available design space. In this approach, each unique unit cell geometry provides a unique design freedom, and when combined, these geometries enable independent control over any two of the following three properties: amplitude, phase, and polarization. The proposed approach also enables any one of these properties to be independently controlled at two different wavelengths. This freedom is used to realize metaoptics with a wide range of functionalities, including multiwavelength holograms, multiwavelength waveplates, and 3D holograms.

**Fig. 1:**
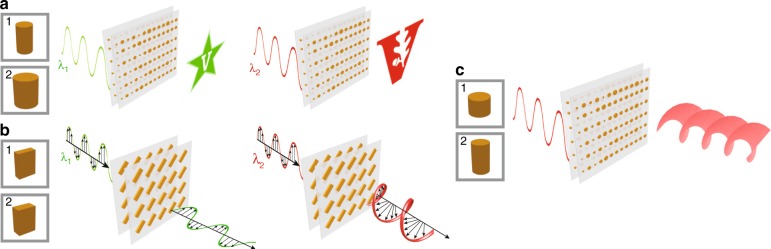
Illustrations of multiwavelength holograms, multiwavelength waveplates, and 3D holograms using bilayer metasurfaces. **a** Illustration of a multiwavelength hologram. The metasurface is designed to achieve independent phase modulation at two wavelengths. **b** Illustration of a multiwavelength waveplate using a combination of two polarization-sensitive rectangular nanopillar geometries. The bilayer metasurface functions as a half-wave plate and a quarter-wave plate at two different, and independent, wavelengths. **c** Schematic of a metaoptic for producing a 3D hologram with on-axis evolution. The bilayer metasurface comprises nanodisks and nanoposts to enable polarization-insensitive control over both amplitude and phase

A schematic of our general approach is shown in Fig. 1. We start with independent control of the phase at two different wavelengths, which is achieved by using nanopost unit cells in both layers (Fig. 1a). The nanopost unit cell enables 2π phase coverage while maintaining high transmission, which, in the bilayer format, can be used to encode independent phase-only holograms at two different wavelengths. Independent control of the polarization at two different wavelengths can be achieved by utilizing polarization-sensitive resonators in both layers (Fig. 1b). This combination can be used to design independent waveplates operating at each wavelength. Finally, the design space can be further enlarged by using two dissimilar resonator geometries. For instance, independent phase and amplitude control can be achieved by combining a nanopost geometry for phase control and a strongly resonant nanodisk geometry for amplitude control, as shown in Fig. 1c. While previous phase-only meta-holograms have suffered from speckle noise and a limited depth of focus, such amplitude and phase holograms enable clearer 2D, as well as 3D, holograms. Furthermore, the use of bilayer metasurfaces enables amplitude and phase control that is independent of the polarization, unlike in single-layer designs, whose operation is restricted to circularly polarized light of a particular handedness^15–17^. We believe that the increased engineering freedom provided by bilayer metasurfaces will open new avenues for the development of a wide range of planar multifunctional metaoptics.

## Results

### Metaoptics for multiwavelength phase control

**Fig. 2:**
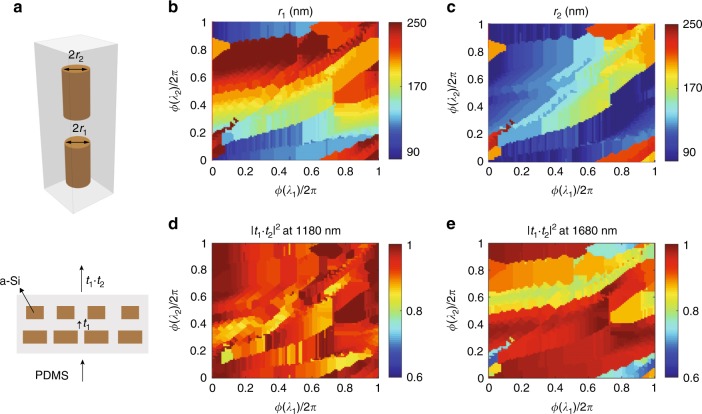
Unit cell and design plots for a multiwavelength metaoptic based on nanoposts. **a** Schematic of a bilayer metasurface unit cell consisting of amorphous silicon nanoposts vertically stacked in close proximity. The nanoposts have a height of 750 nm and are arranged in a square lattice with a period of 600 nm and embedded in a PDMS layer. The transmission coefficient (*t*) of the bilayer is calculated as the product of the transmission coefficients of each individual layer (*t*_1_ and *t*_2_). **b**, **c** Design plots showing the corresponding radii in layer 1 (*r*_1_) and layer 2 (*r*_2_), as indicated by the color bars, for achieving all possible combinations of *ϕ*(*λ*_1_) (the phase at 1180 nm) and *ϕ*(*λ*_2_) (the phase at 1680 nm). **d**, **e** Corresponding transmission values (|*t*_1_*t*_2_|^2^) at 1180 nm **d** and 1680 nm **e**

The proposed bilayer metasurfaces for multiwavelength phase control are based on high-contrast transmit arrays made of silicon (Si) nanoposts with a high aspect ratio. The nanoposts, which can be modeled as truncated waveguides, serve as ideal building blocks for phase modulation due to their high transmission efficiency, localized resonances, and low sensitivity to the angle of incidence^10^. To begin, we explore multiwavelength phase control by cascading two layers of cylindrical nanoposts while keeping them uncoupled in the vertical direction. Figure 2a shows a schematic of the corresponding unit cell embedded in a layer of polydimethylsiloxane (PDMS). The independent variation of the nanopost radii (*r*_1_ and *r*_2_) in each layer provides independent phase control at two different illumination wavelengths. Here, we have arbitrarily selected working wavelengths of 1180 and 1680 nm. The height of the Si nanoposts is 750 nm, and they are arranged in a square lattice with a period of 600 nm. To mitigate wavefront divergence between the layers, the nanoposts are placed in close proximity along the *z* direction but remain uncoupled (Fig. S1), such that the transmission coefficient of the bilayer can be assumed to be the product of the transmission coefficients (*t*_1_ and *t*_2_) of each individual layer. The independent control of the radii in each layer allows one to independently specify the spatial phase profile at each wavelength. Figure 2b and c show the required radii in layer 1 (*r*_1_) and layer 2 (*r*_2_), along with the corresponding transmission values (Fig. 2d and e), for achieving all combinations of *ϕ*(*λ*_1_) (the phase at 1180 nm) and *ϕ*(*λ*_2_) (the phase at 1680 nm); the deviation between the designed and ideal phases is presented in Fig. S2. These design plots can be used to quickly select the structural parameters of each layer to achieve arbitrary and independent phase profiles at the two wavelengths of interest.

**Fig. 3:**
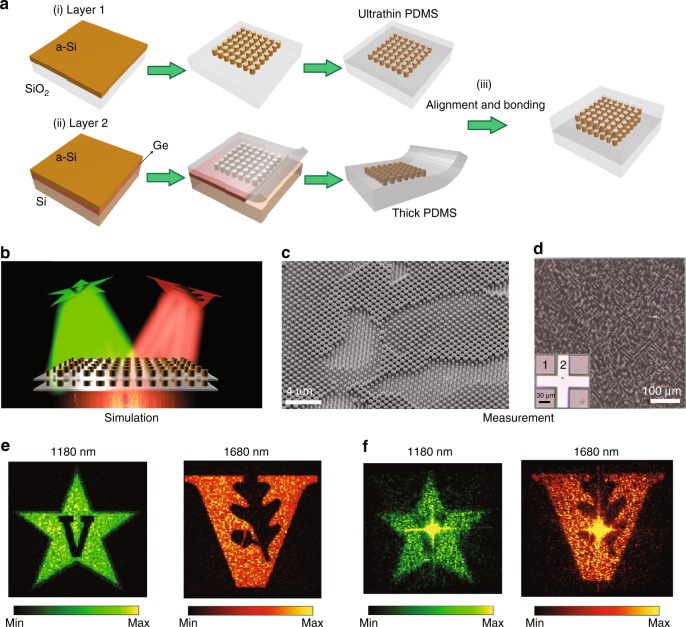
Fabrication and characterization of a multiwavelength holographic metaoptic. **a** Schematic illustration of the fabrication steps. The first layer of Si nanostructures was defined on a silica substrate and embedded in a thin layer of PDMS. The second layer was transferred into PDMS and bonded with the first layer. **b** Schematic of the bilayer hologram consisting of two independent Vanderbilt logos projected at two independent wavelengths. **c** SEM image of the Si nanoposts before PDMS spin coating. **d** Optical images of the bonded metaoptic and the alignment marks in each layer (inset). **e**, **f** Simulated **e** and captured **f** images produced under illumination wavelengths of 1180 and 1680 nm

We have previously used this architecture to realize multiwavelength lenses^25^; here, we extend the approach to the more arbitrary phase profiles found in holograms. A phase-only low-*k* hologram consisting of two different Vanderbilt University logos projected at the wavelengths of 1180 and 1680 nm (Fig. 3b) has been designed using the Gerchberg–Saxton algorithm^26^. The holographic metasurface has a size of 500 μm, and the images are designed to be displayed in the Fraunhofer region (see the design details in Supplementary Section 1). The fabrication involved traditional lithography and etching followed by metasurface transfer and bonding. A schematic illustration of the fabrication flowchart is shown in Fig. 3a. The first layer of the metasurface was defined using electron beam lithography (EBL) and reactive ion etching (RIE) followed by encapsulation in a thin layer of diluted PDMS to allow close stacking of the second layer. The nanostructures of the second layer were created on a 300 nm-thick germanium (Ge) sacrificial layer using the same procedures used for the first layer, followed by release through wet etching of the Ge layer. The second layer was then aligned and bonded to the first layer using a 2D material transfer tool. The details of the fabrication and alignment setups are presented in Supplementary Sections 2 and 3 and Supplementary Fig. S3. Figure 3c shows a scanning electron microscope (SEM) image of the Si nanoposts after RIE. Optical images of the metasurface doublet and the alignment marks in each layer (inset) are shown in Fig. 3d. Both layers are clearly visible under a ×20 optical microscope, indicating a separation smaller than the focal depth of the objective, which is 5.8 μm.

The metasurface was characterized using an unpolarized supercontinuum laser that was passed through a monochromator. To reduce the beam diameter, a lens (*f* = 200 mm) was used to partially focus the beam onto the sample, and the images produced by the metasurface were directly recorded on a near-infrared camera (for the details of the optical systems, see the “Methods” section and Supplementary Fig. S4). To evaluate the design, the transmission of the bilayer metasurface was calculated as a direct product of the designed transmission through each individual layer. The simulated far-field images are shown in Fig. 3e. The absence of unwanted diffraction orders indicates excellent multiwavelength phase coverage. The measured images are presented in Fig. 3f, showing good agreement with the simulations and an absence of twin images, such as those observed in previous work on multiwavelength holograms^21,22^ based on spatially multiplexed metasurfaces. The intense spots in the center are primarily due to the beam overfilling the metasurface, which results in additional zero-order diffraction. The reduced image quality compared with the simulations is attributed to the lateral misalignment of the layers, which is estimated to be 3 μm (see Supplementary Fig. S5). The misalignment tolerance is expected to be improved by employing a larger holographic metasurface, under the assumption of the same maximum diffraction angles.

While it is difficult to gauge the hologram efficiency due to the prohibitively large simulation domain and the beam overfilling in the experiment, we can quantify the diffraction efficiency of similar devices. To accomplish this, a wavelength-multiplexed grating was designed to deflect incident light at 1180 and 1680 nm to −15° and 15°, respectively. The grating has a size of 200 μm, and the metasurfaces are separated by 3 μm (for the details of the simulation, see the Supplementary Methods and Supplementary Fig. S6). The device was simulated using a finite-difference time-domain (FDTD) solver (MEEP)^27^, and the absolute efficiency was calculated as the ratio of the power diffracted into the desired order over the total power incident on the device. The calculated transmission is 81.4% at 1180 nm and 83% at 1680 nm, and the relative diffraction efficiencies are 59% at 1180 nm and 61% at 1680 nm, resulting in absolute efficiencies of 48.1% and 50.3% at 1180 and 1680 nm, respectively. These efficiencies are consistent with the theoretical and experimental efficiencies of multilayer metalenses^25^ based on the same architecture.

### Metaoptics for multiwavelength polarization control

**Fig. 4:**
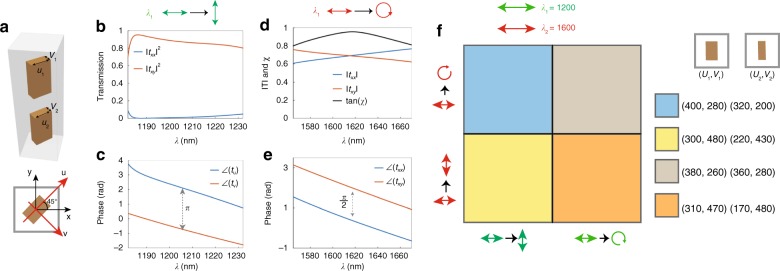
Multiwavelength metaoptic waveplate. **a** Schematic of the rectangular nanopillar unit cell. The metasurface is designed to function as a half-wave plate and a quarter-wave plate at 1200 and 1600 nm, respectively. The resonators have a height of 750 nm and are arranged in a square lattice and oriented at 45° with respect to the *x*-axis. The lengths and widths (*u*, *v*) are (400, 280) and (320, 200) in the bottom and top layers, respectively. **b**, **c** Simulated transmission profiles for the *x* and *y* polarizations **b** and phase delays for the short and long axes **c** at wavelengths near 1200 nm. **d**, **e** Simulated transmission amplitude and ellipticity (tan(*χ*)) profiles **d** and phases **e** for the *x* and *y* polarizations at wavelengths near 1600 nm. **f** Design graph for achieving all combinations of waveplates at the two considered wavelengths. The *x* and *y* axes correspond to the half-wave and quarter-wave plate cases at 1200 and 1600 nm, respectively, and the colors represent the designed nanopillar widths and lengths, as indicated in the legend on the right

The proposed approach can be further extended to achieve independent polarization conversion at multiple wavelengths. To achieve this, polarization-sensitive rectangular nanopillars were selected because their length and width can be independently tuned to achieve polarization-dependent phase retardance. As a proof of concept, we designed a bilayer metasurface that functions as a half-wave plate at a wavelength of 1200 nm and a quarter-wave plate at 1600 nm. Figure 4a shows a schematic of the unit cell of this metasurface. The resonators are oriented at a 45° rotation, and the length and width (*u*, *v*) are designed to be (400, 280) and (320, 200) nm for each layer, resulting in phase retardances between the long and short axes of *π* and *π*/2 for the wavelengths of 1200 and 1600 nm, respectively. Figure 4b and c show the simulated transmission and phase retardances, respectively, at 1200 nm. The metaoptic has a polarization conversion efficiency ($\left| {t_{xy}} \right|^2$) of over 80% and a phase retardance of *π* between wavelengths of 1180 and 1230 nm. Figure 4d and e show the simulated transmission amplitudes and phases, respectively, at 1600 nm for the *x* and *y* polarizations. At 1616 nm, we observe a transmission of 47.5% for both polarizations while maintaining a *π*/2 phase difference, resulting in conversion from linearly to circularly polarized light with 95% efficiency. To numerically determine the polarization state, we extracted the ellipticity, tan(*χ*), by calculating the Stokes parameters (for the details of the calculation, see the “Methods” section), revealing an ellipticity of larger than 0.8 from 1565 to 1670 nm (Fig. 4d). While this functionality could have been achieved using an unstructured material with substantial dispersion, the true advantage of the proposed approach is that any combination of waveplates can be readily realized by modifying the widths and lengths of the nanopillars in each layer. To illustrate this freedom, we present a design graph for achieving all combinations of half-wave and quarter-wave plates at the wavelengths of 1200 and 1600 nm in Fig. 4f (additional details regarding the transmission and ellipticity can be found in Supplementary Fig. S7).

### Metaoptics for polarization-insensitive phase and amplitude control

**Fig. 5:**
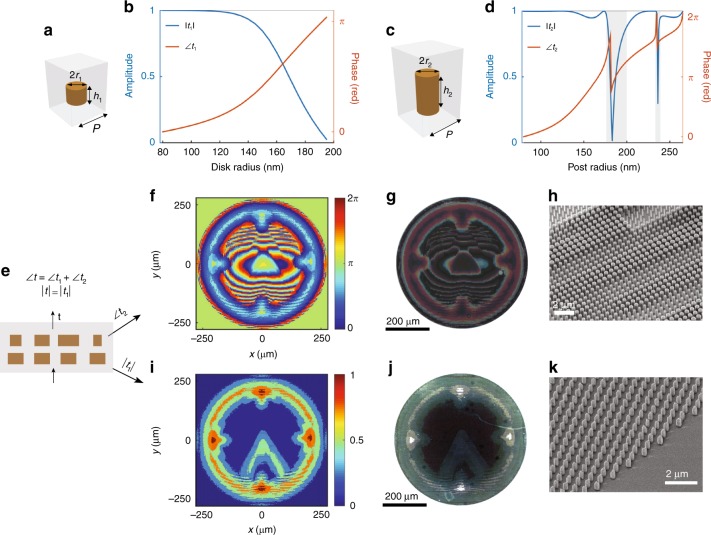
Metaoptic for polarization-insensitive phase and amplitude modulation. **a** Schematic of the Si nanodisk that serves as the unit cell for the bottom layer. The disks have a height of *h*_1_ = 440 nm and are arranged in a square lattice with a period of *P* = 600 nm. **b** Simulated transmission amplitude $\left| {t_1} \right|$ and phase $\angle t_1$ as functions of the disk radius. **c** Schematic of the nanopost that serves as the unit cell for the second layer. The nanoposts have a height of *h*_2_ _=_ 750 nm. **d** Simulated transmission amplitude $\left| {t_2} \right|$ and phase $\angle t_2$ as functions of the post radius. **e** Schematic of the bilayer metasurface. The bottom layer is used for amplitude modulation ($\left| t \right|$), and the top layer is used for phase modulation $(\angle t_2)$. The total phase profile of the bilayer metasurface is $\angle t_1 + \angle t_2.$ **f**, **g**, **h** Designed phase pattern **f**, along with an optical microscope image **g** and an SEM image **h** of the top metasurface. **i** The designed amplitude profile. **j**, **k** Optical **j** and SEM **k** images of the bottom metasurface. All phases and amplitudes are calculated for *λ* = 1400 nm

Finally, we explore the use of the proposed bilayer architecture to achieve complete control over amplitude and phase. In the past amplitude and phase meta-holography, amplitude modulation has been achieved through polarization conversion^15–17^, resulting in an efficiency loss due to polarization sensitivity. Here, we use a bilayer metasurface composed of polarization-insensitive elements for phase and amplitude modulation. Wavefront control is achieved by assigning phase modulation to one layer and amplitude modulation to the other. Amplitude modulation is achieved by utilizing Si nanodisks that support fundamental Mie resonances at the wavelength of operation, and the spectral positions of the resonances can be adjusted by changing the nanodisk radius. In previous work, similar resonators have been used to achieve Huygens surfaces^28^, perfect reflectors^29^, and polarizing beam splitters^13^. Here, we have designed the nanodisks to operate near the magnetic dipole resonance, with a transmission amplitude that is controlled by tuning the structures to work either at or away from the resonance. A schematic of the unit cell is shown in Fig. 5a; the nanodisks have a height and period of 440 and 600 nm, respectively. Figure 5b shows the transmission amplitude and phase as functions of the nanodisk radius at a wavelength of 1400 nm, demonstrating 100% amplitude modulation along with a phase change of *π*. The second layer is used to implement the designed phase functions by means of cylindrical nanoposts with a larger aspect ratio than that of the nanodisks. Figure 5c shows a schematic of the second-layer unit cell, which is used for phase modulation. The nanoposts have a height of 750 nm and a period of 600 nm, and the corresponding transmission amplitude and phase as functions of the nanopost radius are presented in Fig. 5d. The transmission dips highlighted in gray were excluded from the design database, resulting in 2*π* phase coverage and an average transmission of 96%.

As a proof of concept, we designed a 3D hologram (600 μm × 600 μm) to display a clock evolving along the *z*-axis. The design of the holographic plate involves three types of 3D holograms. The two hands of the clock are designed to rotate in opposite directions with different angular speeds along the *z*-axis, while the outer frame remains in focus over an extended depth of view (for the details of the hologram design, see Supplementary Section 1). Figure 5f–h show the designed phase profile (Fig. 5f) along with corresponding optical (Fig. 5g) and SEM (Fig. 5h) images of the nanopost metasurface. The designed amplitude profile is presented in Fig. 5i. Optical and SEM images of the fabricated metasurface are shown in Fig. 5j and k, respectively. The optical images illustrate the substantial difference between the two metasurfaces and indicate that the global distribution of the unit cells is correct. Note that the nanodisks (layer 1) used for amplitude modulation also introduce a phase shift, and thus, the phase profile of the nanopost layer (layer 2) is designed to compensate for this variation.

## Discussion

**Fig. 6:**
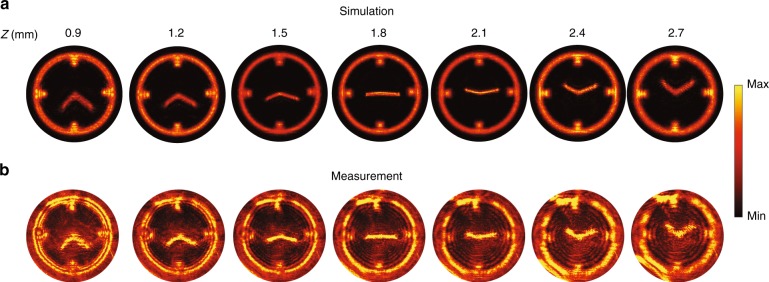
Three-dimensional meta-hologram with on-axis evolution. **a**, **b** Simulated **a** and captured **b** on-axis evolution of the clock hologram under an illumination wavelength of 1330 nm

Figure 6a shows the simulated on-axis evolution of the hologram from *z* *=* 0.9 mm to *z* *=* 2.7 mm based on the designed phase and amplitude patterns from Fig. 5f and i. To determine the performance of the fabricated device, the metasurface was characterized using a collimated and unpolarized monochromatic beam. The incident wavelength was tuned slightly to 1330 nm to achieve the best performance, and image slices were recorded at various distances using a ×20 objective paired with a tube lens (*f* *=* 200 mm). The real images acquired at the various on-axis planes are presented in Fig. 6b and show good agreement with the simulation. It should be noted that the intensity maps have been slightly saturated to reveal clear outlines in the images. The lower contrast and resolution in the measured images are mainly due to the strong structural sensitivity of the nanodisk layer, resulting in errors in the amplitude mask, as illustrated in Fig. S8. To illustrate the role of misalignment, we also realized 3D holograms based on two layers of waveguide-based nanopillars (Fig. S10), which permit polarization-sensitive amplitude and phase control (see Fig. S10 for details). In this case, the structural sensitivity is quite low, and as a result, the far-field images (Fig. S10n) show an absence of background noise and good agreement with the simulation. While better control over the nanodisk size should alleviate the background noise, high-resolution polarization-independent greyscale transmission masks can also be achieved using high-energy beam-sensitive (HEBS) glass^30,31^.

In conclusion, we have demonstrated a bilayer metasurface architecture with various combinations of unit cells to increase the available design space for metaoptics. This approach can be further extended using other sets of unit cell geometries to achieve independent control over other combinations of properties, as demonstrated in Supplementary Figs. S9 and S10. Metasurfaces sensitive to the angle of incidence^32^ could also be incorporated into the platform for applications, such as holographic storage and augmented reality displays. While we have combined independent metasurfaces here, the proposed fabrication techniques can also be utilized to prepare more complex metaoptics with interacting layers, including topology-optimized devices^33^ and bianisotropic metasurfaces^34,35^. Further improvements in performance are also expected if design optimization methods^36–39^ are employed.

## Materials and methods

### Simulation

The transmission coefficient of the array of Si nanoposts was determined using an open-source rigorous coupled-wave analysis (RCWA) solver^40^. The nanoposts, with a height of 750 nm, were arranged in a square lattice with a lattice constant of 600 nm and embedded in a layer of PDMS. The index of the PDMS was set to 1.4, and the refractive index values of the Si (3.55 and 3.47 at 1180 and 1680 nm, respectively) were obtained using ellipsometry. The complex transmission coefficient was calculated using a plane wave at normal incidence, and nanopost radii that corresponded to dips in transmission were excluded from the design database. The same method was used to obtain the transmission coefficients of nanodisks and rectangular nanopillars. The details of the hologram design are presented in Supplementary Section 1.

### Ellipticity calculation

The polarization state of the quarter-wave plate was numerically determined by using the Stokes parameters:

$$\begin{array}{l}S_0 = \left| {t_{xx}} \right|^2 + \left| {t_{xy}} \right|^2\\ S_1 = \left| {t_{xx}} \right|^2 - \left| {t_{xy}} \right|^2\\ S_2 = 2\left| {t_{xx}} \right|\left| {t_{xy}} \right|{\mathrm {cos}}\Delta \varphi \\ S_3 = 2\left| {t_{xx}} \right|\left| {t_{xy}} \right|{\mathrm {sin}}\Delta \varphi \end{array}$$

where *t*_*xx*_ and *t*_*xy*_ are the transmission coefficients for copolarization and cross polarization, respectively, and Δ*φ* is their phase difference, $\angle t_{xy} - \angle t_{xx}$. The ellipticity *χ* can be expressed in terms of the Stokes parameters as follows: $\sin \left( {2\chi } \right) = \frac{{S_3}}{{S_0}}$.

### Measurement

The multiwavelength hologram was characterized using the setup shown in Fig. S4a. The samples were illuminated using a collimated supercontinuum laser (Fianium WhiteLase) that was passed through a monochromator (Cornerstone^TM^ 130 1/8 m). To reduce the beam size, a lens (AC254-200-C-ML, *f* = 200 mm) was placed in front of the device to partially focus the light. The far-field hologram images were recorded using an InGaAs NIR camera (Xeva-1.7-640).

The 3D clock hologram was characterized using a custom imaging system with a ×20 objective (Mitutoyo Plan Apo, NA = 0.4) paired with a tube lens (*f* = 200 mm). The device was mounted on a translation stage and moved along the axial direction to measure the on-axis evolution of the hologram, as shown in Fig. 6b. A schematic of the characterization system is shown in Fig. S4.

## Supplementary information

Supplementary Information
