# Supplementary material for: Multifunctional metaoptics based on bilayer metasurfaces
Source: Light Sci Appl. 2019 Sep 4;8:80. doi: 10.1038/s41377-019-0193-3 (PMC6804919; doi:10.1038/s41377-019-0193-3)
Supplement: Supplementary file 1 — Supplementary Information [file 41377_2019_193_MOESM1_ESM.pdf]

Supplementary Information for: Multifunctional Metaoptics Based on Bilayer Metasurfaces

You Zhou[1], Ivan I. Kravchenko[2], Hao Wang[3], Hanyu Zheng[4], Gong Gu[3] and Jason Valentine[5*]

[1]Interdisciplinary Materials Science Program, Vanderbilt University, Nashville, Tennessee 37212, USA
[2]Center for Nanophase Materials Sciences, Oak Ridge National Laboratory, Oak Ridge, Tennessee 37831, USA
[3]Min H. Kao Department of Electrical Engineering and Computer Science, University of Tennessee, Knoxville, Tennessee 37996, USA
[4]Department of Electric Engineering and Computer Science, Vanderbilt University, Nashville, Tennessee 37212, USA
[5]Department of Mechanical Engineering, Vanderbilt University, Nashville, Tennessee 37212, USA
[*]email: jason.g.valentine@vanderbilt.edu

Section 1: Multiwavelength hologram design

The multiwavelength hologram was designed using a brute search algorithm. The transmission properties of the bilayer metasurface were calculated using the product of the transmission coefficients from each layer,

$$t(\lambda, r_1, r_2) = t_1(\lambda, r_1, r_2) \cdot t_2(\lambda, r_1, r_2)$$

where r_1 and r_2 are the radii of the nanoposts at each layer. The phase patterns for producing different hologram images were calculated using the Gerchberg-Saxton algorithm[1], resulting in two independent target phase masks (t_{λ_1} and t_{λ_2}) for each wavelength. At each lattice size, the radii at each layer (r_1, r_2) were designed by calculating the total error from the desired transmission masks at the two wavelengths

$$\Delta(x, y) = \left|t_{\lambda_1}(x, y) - t(\lambda_1, r_1, r_2)\right| + \left|t_{\lambda_2}(x, y) - t(\lambda_2, r_1, r_2)\right|$$

The transmission coefficients of the metasurface for each wavelength were then selected based on the designed nanoposts at each layer, and the far-field images were calculated by taking a Fourier

transform of the transmission masks.

The full wave simulation of the grating in Fig. S6 was carried out using an open source FDTD solver (Meep[2]). A multiwavelength blazed grating was designed to diffract the incident light at 15° and -15° at the wavelengths of 1180 and 1680 nm, respectively. The bilayer metasurface was embedded in a layer of PDMS and simulated with a 3 μm vertical layer spacing. The transmitted and total incident power were acquired by using a Gaussian pulse source and measuring the power flux with and without the device. The relative diffraction efficiency was calculated using a plane wave source to acquire the complex electric field after the metasurface and the diffracted power was obtained by taking its Fourier transform. The absolute diffraction efficiency was calculated using the product of the transmission and relative diffraction efficiency.

3D hologram design

To design a 3D clock hologram with on-axis evolvement, three types of 3D holograms were created that comprise the frame and two hands of the clock. The frame is composed of a 3D hollow cylinder with an extended depth of focus from z=0.9 mm to 2.7 mm. To reduce on-axis image distortion, the ring image was sampled into multiple slices along z axis and a constant phase distribution $k \times z$ was added to each xy-plane, where k is the wave vector in free space. The transmission masks were then used to propagate a beam backwards at the wavelength of 1400 nm by the distance away from the metasurface, and the complex field profiles (E_{frame}) were calculated by the sum of contributions from all slices. The central areas with amplitude smaller than 0.2 were set to zero for incorporating additional hologram design.

To design the two hands of the clock, two rectangular amplitude masks with different length (L_1=35 μm, L_2=73 μm) were created to appear at the distance of 1.8 mm away from the

metasurface. A phase pattern was added to each rectangle for generating an angular velocity, which is given by $\varphi = \omega r^2\theta$. Here, r and θ are the radius and angle in polar coordinates. Thus, in Cartesian coordinates the complex electric field profile for creating the hands of clock is

$$E_{han}\ (x,y) = E_{rect}^{1}(x,y)\cdot \exp[i\omega_1(x^2+y^2)\ arctan\frac{y}{x}] + E_{rect}^{2}(x,y)\cdot \exp[i\omega_2(x^2+y^2)\ arctan\frac{y}{x}]$$

where E_{rect}^{1} and E_{rect}^{2} are the amplitude profiles of the two rectangles. Independent angular velocities of $\omega_1 = 5.2\times10^{-3}$ μm^{-2} and $\omega_2 = -3.6\times10^{-3}$ μm^{-2} were added to each amplitude mask. The field was then used to back propagate a beam for a distance of 1.8 mm to generate the phase and amplitude of the metasurface. To increase the misalignment tolerance, every pixel on the metasurface is expanded to a 4×4 supercell. Combined with the field distribution for the clock frame, the total electric field profile is,

$$E(x,y) = E_{frame}(x,y) + E_{hand}(x,y)$$

Section 2: Fabrication

The first layer of the metasurface was defined on 750 thick a-Si, which was grown on a SiO_2 substrate by plasma-enhanced chemical vapor deposition (PECVD). A 200 nm PMMA A4 layer was spin coated at 4500 rpm, followed by deposition of a 10 nm thick chromium conduction layer using thermal evaporation. The metasurface structures were then defined using electron beam lithography (EBL), followed by a 35 nm thick alumina oxide deposition as a dry etch mask using e-beam evaporator. The Si nanoposts were then formed by reactive ion etching in a mixture of C_4F_8 and SF_6. To completely encapsulate the nanoposts, PDMS (10:1 mixing ratio of Sylgard 184 base and curing agent) was diluted with toluene in a 2:3 weight ratio, which was then spin coated on the substrate at 2500 rpm and cured at 80 C° for more than half an hour.

The substrate for patterning the second metasurface layer was prepared by first depositing a 300 nm germanium (Ge) layer on a Si handle wafer using Ar sputtering from a 3” Ge target, followed by growing the same thickness a-Si using PECVD. A second metasurface was defined using the same procedures as the first followed by encapsulation by spin coating a diluted PDMS solution at 2500 rpm. To form a mechanical support for the transfer, a second layer of PDMS (~50 μm) without dilution (10:1 mixing ratio of Sylgard 184 base and curing agent) was subsequently spin coated at 1000 rpm, followed by curing at 80 C° for more than two hours. The metasurface was then released from the substrate by dissolving the germanium sacrificial layer using a mixture of hydrogen peroxide, ammonium hydroxide and DI water.

Section 3: Alignment

A schematic of the alignment setup is shown in Fig. S3[3]. The first layer of the metasurface was attached on a glass slide and mounted on an XYZ translation stage with the pattern side facing down. The second device was flipped over and placed underneath with the rod side facing up, which was mounted on a ~250 nm thick PMMA held on a copper hot plate using a vacuum pump. To see through the alignment marks from the top, an imaging system was built up using a digital camera (Nikon D3200) paired with a zoom lens (MVL12X3Z) and an extension tube (MVL20FA). The top layer metasurface was brought close to the bottom until both layers are clearly visible, and the alignment was carried out by tuning the rotation and translation of the alignment marks. After the spatial alignment was achieved, the top metasurface stage was lifted up, and a drop of uncured PMDS was applied on the bottom layer. The top metasurface was then brought down to engage with the bottom and squeeze the uncured PDMS until both alignment marks were clearly visible. The two layers were bonded together by turning on the hotplate at 80 C° for more than one hour.

During the PDMS curing process, the translation stage was slightly adjusted to compensate for the thermal drift and system vibrations.

References

1. Gerchberg, R. W. & Saxton, W. O. A Practical Algorithm for the Determination of Phase from Image and Diffraction Plane Pictures. *Optik (Stuttg).* **2,** 237–246 (1969).
2. Oskooi, A. F. *et al.* Meep: A flexible free-software package for electromagnetic simulations by the FDTD method. *Comput. Phys. Commun.* **181,** 687–702 (2010).
3. Castellanos-Gomez, A. *et al.* Deterministic transfer of two-dimensional materials by all-dry viscoelastic stamping. *2D Mater.* **1,** 011002 (2014).

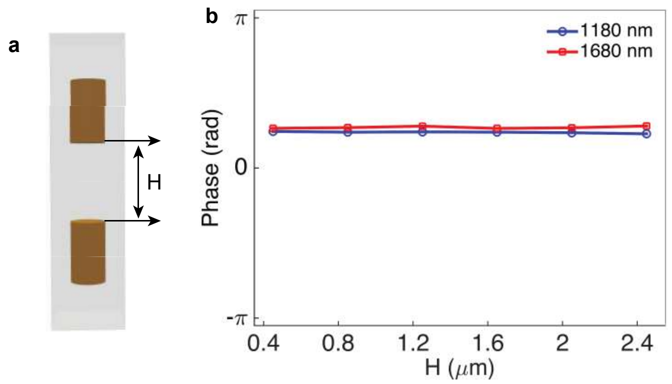

Fig. S1|Phase delay VS layer spacing. (a, b) Simulated phase of the bi-layer unit cell as a function of layer distance H. The constant phase profiles indicate the response of each layer is independent of the other.

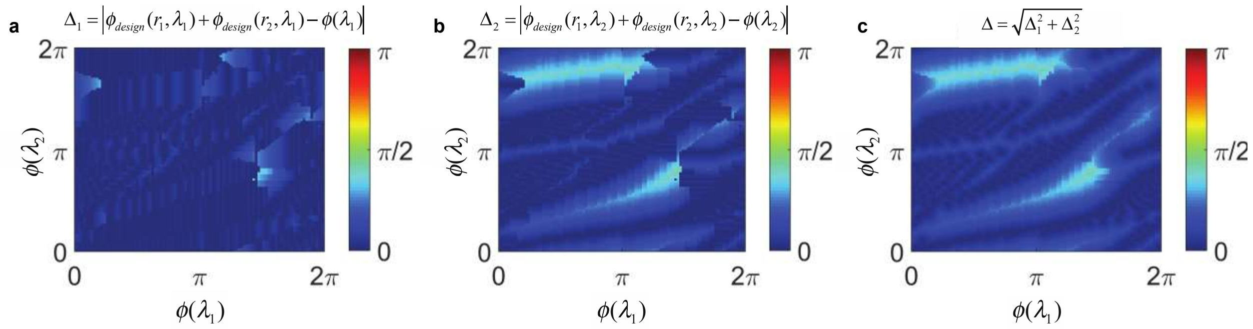

Fig. S2| Color-coded deviation plot between the designed and ideal transmission phase. (a-b) The deviation plot for λ_1 (a) and λ_2 (b). The deviation is defined as $\Delta = \left|\phi_{design}(r_1, \lambda_{1,2}) + \phi_{design}(r_2, \lambda_{1,2}) - \phi(\lambda_{1,2})\right|$ for each wavelength where $\phi(\lambda_{1,2})$ is the desired phase for $\lambda_{1,2}$, respectively. (c) The total deviation plot for both wavelengths.

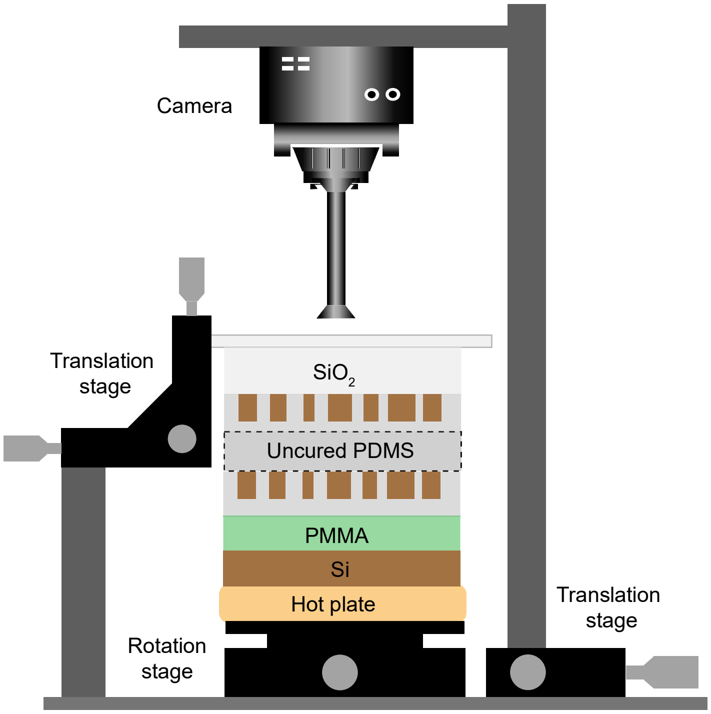

Fig. S3| Schematic of the alignment and bonding processes. A camera paired with a zoom lens and an extension tube was positioned to image through the bilayer metasurface doublet. The bottom metasurface was mounted on a layer of PMMA spin coated on a Si substrate held on a copper hot plate. The top metasurface was attached to a glass slide attached to an XYZ translation stage. Uncured PDMS was applied in between as a thin bounding and index-matched layer.

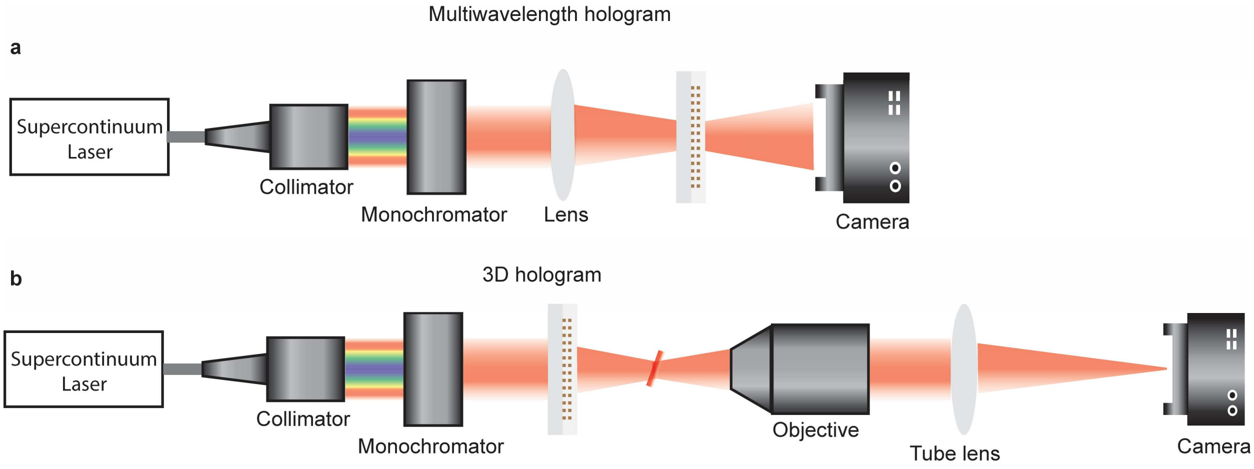

Fig. S4| Schematic of the measurement setup for multiwavelength and 3D hologram characterization. (a) A collimated laser beam was passed through a monochromator and a lens was used to partially focus the beam on the metasurface. The hologram image was directly recorded by a NIR camera. (b) The metasurface was illuminated by a collimated laser beam and the on-axis image slices were captured through an objective, paired with a tube lens, on a camera.

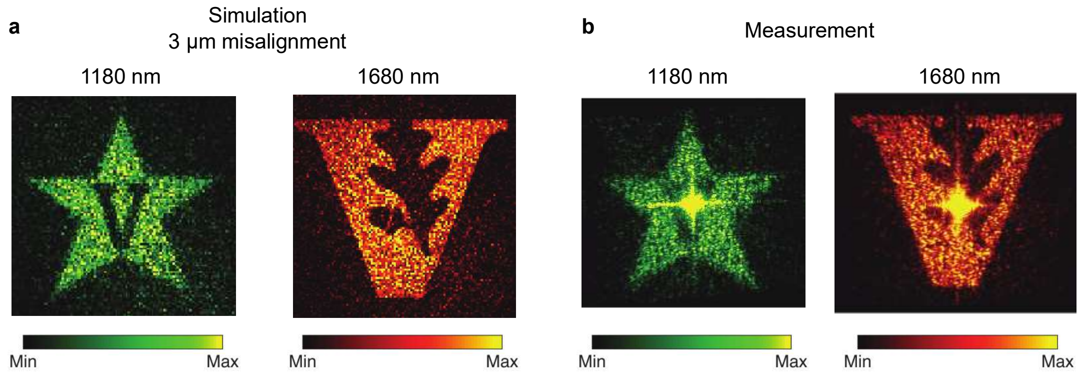

Fig. S5|Measurement compared to a 3 μm metasurface misalignment. Simulated hologram images (a) under a 3 μm misalignment and measurement (b).

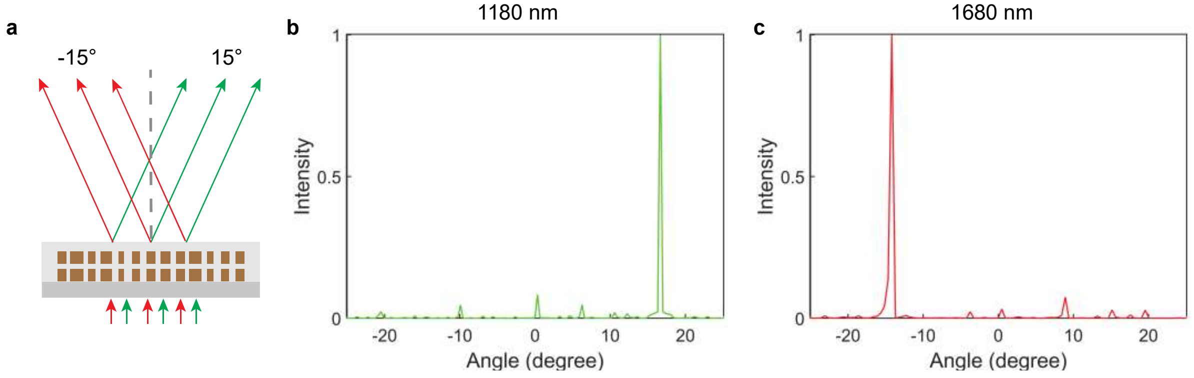

Fig. S6|Wavelength-multiplexed blazed grating. (a) Schematic of the grating that deflects incident light at 1180 and 1680 nm into beams at 15° and -15°. (b-c) Simulated far-field intensity as a function of diffraction angle at 1800 nm (b) and 1680 (c). The absolute diffraction efficiencies are 48.1% and 50.3% at 1180 and 1680 nm, respectively.

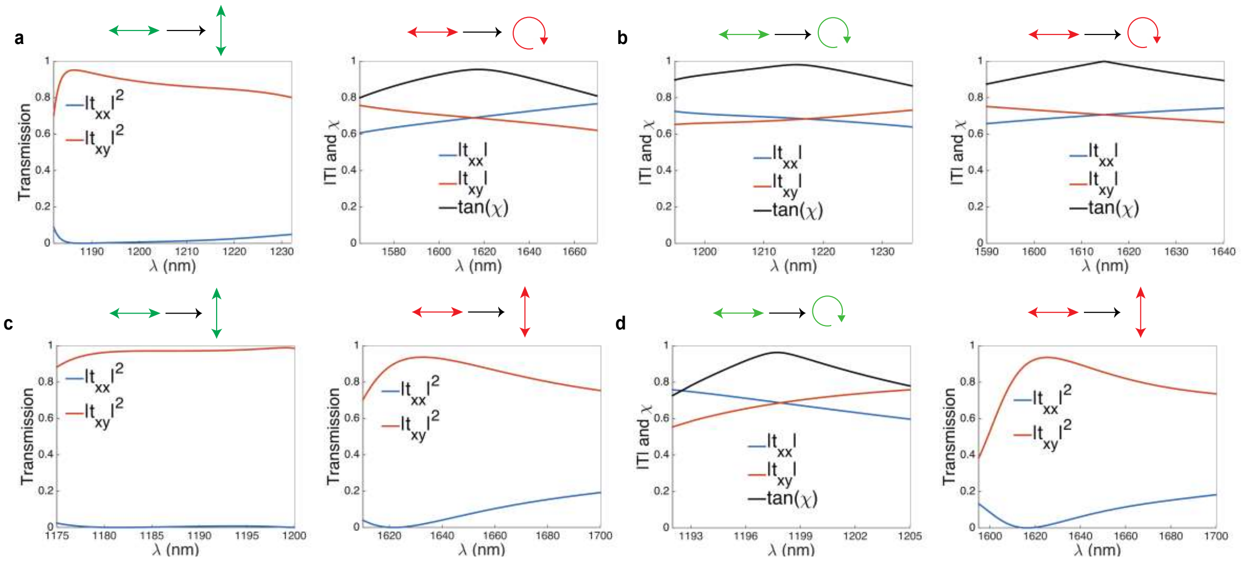

Fig. S7|All combinations of multiwavelength metaoptic waveplate based on polarization sensitive nanopillars. (a-d) Simulated transmission for a HWP at 1200 nm, QWP at 1600 nm (a), QWP at 1600 nm, QWP at 1200 nm (b), HWP at 1200 nm, HWP at 1600 nm (c), and QWP at 1200 nm, HWP at 1600 nm (d), where HWP denotes a half-wave plate and QWP denotes a quarter-wave plate.

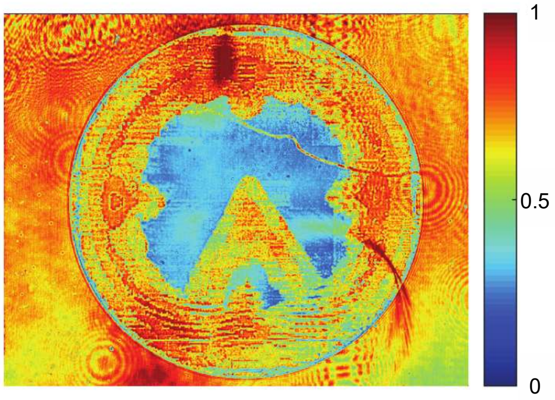

Fig. S8|Transmission map of the amplitude mask.

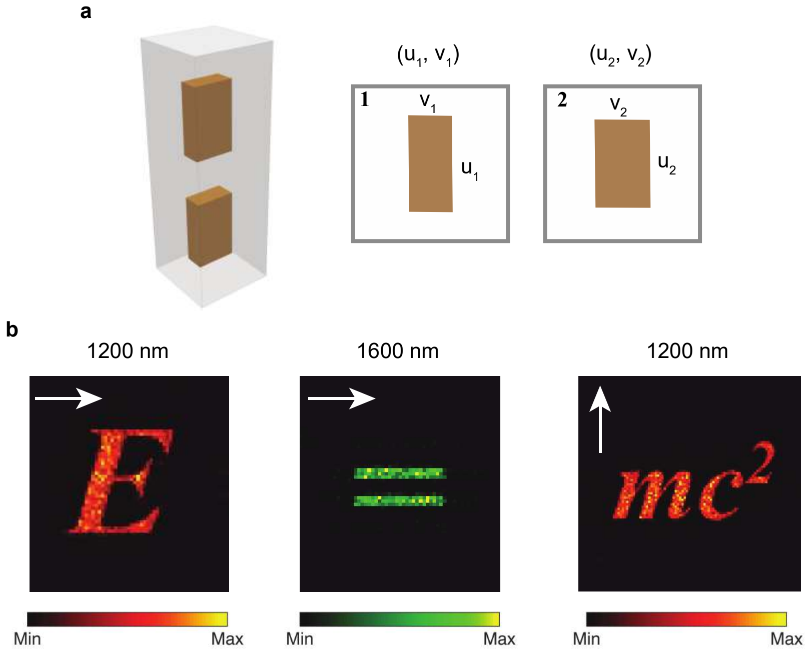

Fig. S9|Multiwavelength metaoptic holograms based on polarization sensitive nanopillars. (a) Schematic of the unit cell which is composed of bilayer rectangular pillars with a height of 750 nm and a period of 600 nm. The unit cell width and length on each layer was adjusted independently for encoding addition information at the two wavelengths. (b) Simulated hologram images. The metasurface is designed to encode two hologram images at two wavelengths under *x* polarization, and a third image for *y* polarization illumination.

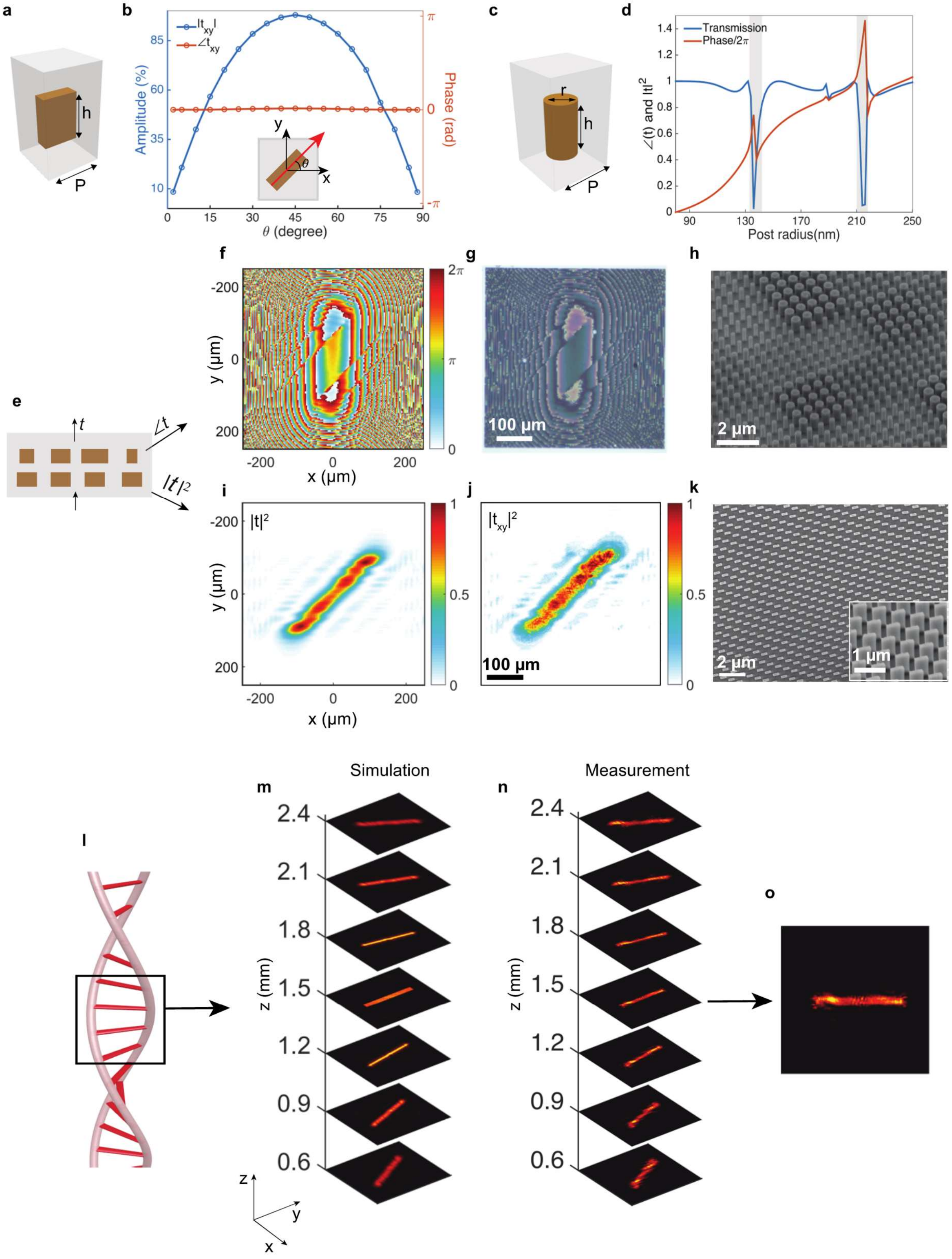

a
b
c
d
e
f
g
h
i
j
k
l
m
n
o
Amplitude (%)
Phase (rad)
θ (degree)
Transmission
Phase/2π
Post radius(nm)
y (μm)
x (μm)
100 μm
2 μm
1 μm
Simulation
Measurement
z (mm)

Fig. S10|Metaoptic for complete amplitude and phase modulation based on linear polarization conversion. (a) Schematic of the first-layer unit cell made of Si rectangular nanopillars. The pillars have a height of 750 nm, a width of 90 nm and a length of 230 nm and are arranged in a square lattice with a period of 600 nm. (b) Simulated cross-polarization conversion amplitude $|t_{xy}|$ and phase $\angle t_{xy}$ as a function of the orientation angle θ with respect to the *x* axis. (c) A schematic of the unit cell on the second layer. The unit cell is a polarization insensitive cylindrical nanoposts with a height of 750 nm. (d) Simulated transmission and phase as a function of post radius. (e) Schematic of the bilayer metasurface. The bottom layer is used for amplitude modulation ($|t|$), and the top layer is used for phase modulation $(\angle t)$. (f, g, h) The designed phase pattern (f), along with an optical microscope (g) and SEM image (h) of the fabricated metasurface. (i, j, k) The bottom metasurface layer for amplitude modulation. (i) Simulated amplitude map and the (j) experimental polarization conversion map $|t_{xy}|^2$. The SEM image of the bottom layer is shown (k). (l-n) (l) A schematic of the 3D hologram with on-axis rotation. The metasurface is designed to show a portion of the helicoid image ranging from *z*=0.6 mm to *z*=2.4 mm. (m, n) Simulated (m) and measured (n) on-axis evolvement of the hologram. In the measurement, two orthogonal polarizers were used before and after the device. (o) Measured image at *z*=1.5 mm, showing the absence of speckle noise.
